# Prasugrel versus ticagrelor for acute coronary syndrome patients undergoing percutaneous coronary intervention: A critical appraisal of randomized controlled trials

**DOI:** 10.1016/j.amsu.2022.103330

**Published:** 2022-02-07

**Authors:** Talal Almas, Maryam Ehtesham, Janita Basit, Tarek Khedro, Uzair Malik, Vikneswaran Raj Nagarajan, Jung Hur, Norah Alshareef, Areen Fathima, Hafeez Ul Hassan Virk, Aamir Hameed, Jun Li

**Affiliations:** a RCSI University of Medicine and Health Sciences, Dublin, Ireland; b University Hospital, National University of Ireland Galway, Galway, Ireland; c Cardiology, Adena Regional Medical Center, Chillicothe, OH, USA; d Tissue Engineering Research Group, Department of Anatomy and Regenerative Medicine, RCSI University of Medicine and Health Sciences, Dublin, Ireland; e Harrington Heart and Vascular Institute, University Hospitals Cleveland Medical Center, Cleveland, OH, USA

Every year, millions of coronary stenting procedures are performed for the treatment of ischemic heart disease. Initially, bare metal stents (BMS) were utilized for acute coronary syndromes (ACS) and myocardial infarctions (MIs). While effective, rates of restenosis with BMS were a concern, subsequently leading to the advent of drug-eluting stents (DES). DES were coated with polymers that enabled the slow and controlled release of anti-proliferative agents, such as sirolimus and paclitaxel [1]. Studies thereafter showed that DES indeed led to a marked decrease in the restenosis rate of stented coronary vessels [2]. Nevertheless, despite the plummeting restenosis rates, thrombosis remained an issue, even in DES, where one of the primary concerns was that their non-biodegradable polymers remained after completing drug release [2,3]. Thereafter, numerous clinical trials and studies have unequivocally demonstrated that treatment with dual antiplatelet therapy (DAPT), a combination of a P2Y12 antagonist and aspirin, is the go-to strategy for these patients [4].

DAPT remains the standard therapy for patients with ACS undergoing percutaneous coronary intervention (PCI) by virtue of DAPT's effectiveness in lowering the risk of ischemic events [4]. Although clopidogrel has been the most commonly used P2Y12 antagonist in ACS patients for over a decade, novel third generation P2Y12 inhibitors, namely ticagrelor and prasugrel, have actually been shown to elicit more robust antiplatelet effects [5,6]. Several randomized control trials have demonstrated superior clinical outcomes of both ticagrelor and prasugrel compared to their predecessor, clopidogrel [5,6]. However, whether ticagrelor or prasugrel have a better comparative efficacy and safety profile in ACS patients who have yet to undergo PCI remains elusive. The lack of consensus with respect to whether ticagrelor or prasugrel should be employed in ACS patients can be attributed to the absence of randomized direct comparison of the drugs' safety and efficacy, a debate that has been addressed by studies attempting to bridge these gaps [[7], [8], [9], [10]].

The Intracoronary Stenting and Antithrombotic Regimen: Rapid Early Action for Coronary Treatment (ISAR-REACT) 5 trial, conducted by Schüpke et al., involved 4018 patients with ACS (41% with ST-segment elevation MI [STEMI]) who had yet to undergo PCI [7]. The authors reported that among patients who presented with ACS with or without ST-segment elevation, prasugrel was superior to ticagrelor in reducing the first-year incidence of primary end-point events, death, MI, and stroke [7]. Of the 2012 patients on ticagrelor, 184 (9.3%) experienced a primary end-point event. Comparatively, out of 2006 patients on prasugrel, 137 (6.8%) experienced a primary end-point event (*p* = 0.006) [7]. The difference of 2.5% was attributed to a lower incidence of MIs in the prasugrel group [7]. Furthermore, they reported no statistically significant incidence of major bleeding in both groups [7]. Thus, prasugrel's observed therapeutic benefit (lower rates of ischemic events) did not come with the cost of increased risk of bleeding. Interestingly, prasugrel's side-effect profile also appears to be generally more favorable compared to that of ticagrelor, primarily with respect to dyspnea. Patients on ticagrelor were more likely to experience dyspnea post-discharge (n = 44 vs n = 1), consequently leading to more frequent discontinuation of ticagrelor than prasugrel. All other side-effects leading to non-adherence to either regimen post-discharge appear to be concordant [7]. However, in the context of any discussions pertaining to side-effect profiles between the two groups, it is worth noting that the reported side-effect profile of prasugrel may be influenced by the higher number of patients who were excluded from safety endpoint analysis in the prasugrel group (n = 233) versus ticagrelor (n = 23) [7]. While the ISAR-REACT 5 trial is limited by its open-label study design and follow-up conducted through telephone contact, the finding that a prasugrel-based strategy is superior in patients presenting with ACS with or without ST-segment elevation is imperative [7]. Furthermore, a post-hoc analysis of this trial by Valina et al. combined the non-STEMI (NSTEM) and unstable angina (UA) subgroups in order to further elucidate the supremacy of prasugrel over ticagrelor in patients undergoing PCI [8]. The authors reported that the primary endpoint was reached in 101 (8.7%) patients on ticagrelor and 73 (6.3%) on prasugrel (hazard ratio [HR]:1.41; 95% confidence interval [CI]: 1.04 to 1.90). Accordingly, the patients on prasugrel had a significant reduction in the combined 1-year risk of death, MI, and stroke without an increase in the risk of bleeding [8].

Motovska et al. conducted the Multicenter Randomized PRAGUE-18 Study intending to compare the clinical efficacy and safety of prasugrel and ticagrelor [9]. The study randomised 1230 patients, planned for PCI, to either ticagrelor or prasugrel. The incidence of primary end-points––defined as death, reinfarction, urgent target vessel revascularization, stroke, serious bleeding, or prolonging hospitalization [9]––was assessed after 7 days. No significant difference was observed between ticagrelor and prasugrel (4.0% vs. 4.1%, *p* = 0.939) [9]. Incidence of secondary endpoints––defined as cardiovascular events, non-fatal MI, or stroke [9]––was assessed after 30 days, again demonstrating no significant difference between prasugrel and ticagrelor (2.7% vs 2.5%, *p* = 0.864) [9]. It is also noteworthy that due to economic constraints, 34.1% of patients on prasugrel and 44.4% of patients on ticagrelor switched to clopidogrel after discharge, further obscuring the true comparative efficacy [9]. The difference in the composite efficacy endpoint at the 1-year follow up study period was similarly insignificant between prasugrel and ticagrelor (6.6% vs. 5.7%, *p* = 0.503), further compounding the esoterism of the therapeutic conundrum [10]. The results from the major clinical trials, stratified with reference to primary and secondary endpoints, are delineated by Table 1 below.

**Table 1:** Comparative efficacy of ticagrelor and prasugrel in major clinical trials with pertinence to primary and secondary endpoints.

End points	Motovska et al., 2016^9^ N (%)		Motovska et al., 2018^10^ N (%)		Schüpke et al., 2019^7^ N (%)	
	Ticagrelor	Prasugrel	Ticagrelor	Prasugrel	Ticagrelor	Prasugrel
Mortality (due to reinfarction, urgent revascularization, stroke, or serious bleeding requiring transfusion or prolonging hospital stay)	25 (4.0)	24 (4.1)				
Primary endpoint: death MI, Stroke					184 (9.3)	137 (6.9)
All-cause mortality	8 (1.3)	12 (2.0)	25 (4.2)	30 (4.7)	90 (4.5)	73 (3.7)
Reinfarction	6 (1.0)	4 (0.7)				
Urgent Revascularization	9 (1.4)	7 (1.2)				
Stroke	1 (0.2)	1 (0.2)				
Serious bleeding requiring transfusion or prolonging hospital stay	8 (1.3)	7 (1.2)				

Similarly, the Motovska et al., 2016 trial, the results of which are depicted in Fig. 1 below, demonstrated a superior side-effect profile for prasugrel with regards to mortality due to reinfarction, urgent revascularization, stroke, or bleeding [9]. Interestingly, within the same trial, ticagrelor was noted to boast a greater efficacy in terms of all-cause mortality due to causes other than those listed [9].

**Fig. 1:**
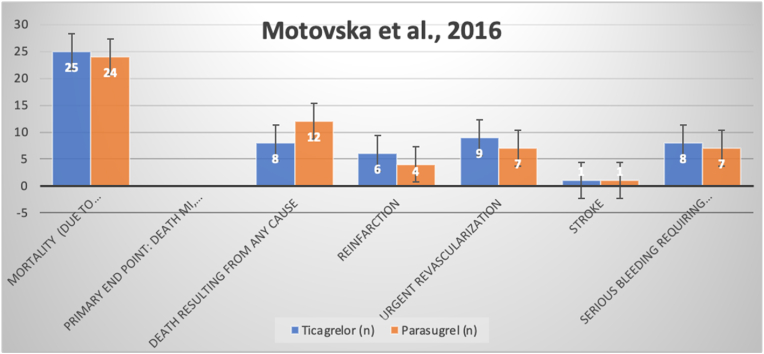
Comparative efficacy of prasugrel and ticagrelor with pertinence to primary endpoints in the Motovska et al., 2016 trial.

## Future perspectives

Although the choice between prasugrel and ticagrelor remains at the epicenter of an ongoing cardiology conundrum, there is paucity of data comparing these two in patients requiring triple therapy, such as the regimens classically employed in atrial fibrillation and deep vein thrombosis patients. Nevertheless, some salient conclusions can be reliably drawn. Firstly, ticagrelor appears to be the preferred drug for upfront loading in ACS patients whereas those undergoing treatment with prasugrel ought to wait for angiography prior to prasugrel loading. However, in less compliant patients, prasugrel remains the preferred antiplatelet of choice due to its once-daily dosing as compared to the twice-daily ticagrelor regimen. While ticagrelor has been studied as a single, monotherapy agent for ACS patients in the MASTER DAPT and GLOBAL Leaders investigator trials, the data for prasugrel monotherapy is less august [11,12]. In order to better inform the debate on what should constitute the optimal antiplatelet agent, both the drugs should be studied in a head-to-head, comparative fashion in stable ACS patients undergoing PCI.

## Ethical approval

NA.

## Sources of funding

NA.

## Author contribution

TA, MA, JB: wrote the abstract, case presentation, study concept, design, conclusion, limitation.

TK, UM, VRN: wrote the introduction.

JH, NA, AF, HUHV: revised the edits, figures, chart review.

TA, HUHV, AH, JL: performed the final edits, critically revised the paper, and gave final approval.

## Consent

NA.

## Registration of research studies

Name of the registry: NA.

Unique Identifying number or registration ID: NA.

Hyperlink to your specific registration (must be publicly accessible and will be checked): NA.

## Guarantor

Talal Almas RCSI University of Medicine and Health Sciences123 St. Stephen's GreenDublin 2, Ireland. Talalalmas.almas@gmail.com.

## Declaration of competing interest

The authors declare no conflict of interest.
